# Oral versus intravenous antibiotics for patients with *Klebsiella pneumoniae* liver abscess: study protocol for a randomized controlled trial

**DOI:** 10.1186/1745-6215-14-364

**Published:** 2013-10-31

**Authors:** James Molton, Rachel Phillips, Mihir Gandhi, Joanne Yoong, David Lye, Thuan Tong Tan, Dale Fisher, Sophia Archuleta

**Affiliations:** 1 Division of Infectious Diseases, University Medicine Cluster, National University Health System, Singapore, Singapore; 2 Department of Medicine, Yong Loo Lin School of Medicine, National University of Singapore, Singapore, Singapore; 3 Singapore Clinical Research Institute, Singapore, Singapore; 4 Saw Swee Hock School of Public Health, National University of Singapore, Singapore, Singapore; 5 Department of Infectious Diseases, Communicable Disease Centre, Tan Tock Seng Hospital, Singapore, Singapore; 6 Department of Infectious Diseases, Singapore General Hospital, Singapore, Singapore

**Keywords:** Klebsiella pneumoniae, Liver abscess, Antibiotics, Ceftriaxone, Ciprofloxacin, Outpatient, Randomized-controlled trial, Non-inferiority trial, Healthcare economics

## Abstract

**Background:**

*Klebsiella pneumoniae* liver abscess is the most common etiology of liver abscess in Singapore and much of Asia, and its incidence is increasing. Current management includes prolonged intravenous antibiotic therapy, but there is limited evidence to guide oral conversion. The implicated K1/K2 capsule strain of *Klebsiella pneumoniae* is almost universally susceptible to ciprofloxacin, an antibiotic with high oral bioavailability. Our primary aim is to compare the efficacy of early (< one week) step-down to oral antibiotics, to continuing four weeks of intravenous antibiotics, in patients with Klebsiella liver abscess.

**Methods/design:**

The study is designed as a multi-center randomized open-label active comparator-controlled non-inferiority trial, with a non-inferiority margin of 12%. Eligible participants will be inpatients over the age of 21 with a CT or ultrasound scan suggestive of a liver abscess, and *Klebsiella pneumoniae* isolated from abscess fluid or blood. Randomization into intervention or active control arms will be performed with a 1:1 allocation ratio. Participants randomized to active control will receive IV ceftriaxone 2 grams daily to complete a total of four weeks of IV antibiotics. Participants randomized to intervention will be immediately converted to oral ciprofloxacin 750 mg twice daily. At Week four, all participants will undergo abdominal imaging and be assessed for clinical response (CRP < 20 mg/l, absence of fever, plus scan showing that the maximal diameter of the abscess has reduced). If criteria are met, antibiotics are stopped; if not, oral antibiotics are continued, with reassessment for clinical response fortnightly. If criteria for clinical response are met by Week 12, the primary endpoint of clinical cure is met. A cost analysis will be performed to assess the cost saving of early conversion to oral antibiotics, and a quality of life analysis will be performed to assess whether treatment with oral antibiotics is less burdensome than prolonged IV antibiotics.

**Discussion:**

Our results would help inform local and international practice guidelines regarding the optimal antibiotic management of Klebsiella liver abscess. A finding of non-inferiority may translate to the wider adoption of a more cost-effective strategy that reduces hospital length of stay and improves patient-centered outcomes and satisfaction.

**Trial registration:**

Clinical trials gov NCT01723150

## Background

While *Klebsiella pneumoniae* is an emerging cause of community acquired liver abscess in Asian populations, there are no clear guidelines on appropriate management. Klebsiella liver abscess (KLA) syndrome differs from other pyogenic liver abscesses in that patients tend to have a normal biliary system and poorly controlled diabetes is a predisposing factor. The increased incidence in Asia is likely explained by the prevalence of the K1 and K2 capsular serotype in this region. These virulence factors possessed by *K. pneumoniae* are thought to protect from phagocytosis by neutrophils, and are associated with septic metastatic complications to brain and eye [1]. In Singapore, KLA has become the most common type of liver abscess [2,3]. In Taiwan, a retrospective study of 182 cases of pyogenic liver abscess included 160 cases of *K. pneumoniae* monoinfection [4]. All 160 *K. pneumoniae* isolates were resistant only to ampicillin, and sensitive to cephalosporins (typical of the community acquired *K. pneumoniae* strain). Of the *K. pneumoniae* patients, 67.5% had frank diabetes and an additional 7.5% had impaired glucose tolerance. Only 0.6% had intraductal stones, and none had intra-abdominal pathology. This contrasts sharply with the 77.3% of the polymicrobial abscess patients with intraductal stones and 18.2% with intra-abdominal malignancy. Mortality from KLA was 11.3%, and relapse was 4.4%.

Management of KLA consists of antibiotics, and in most instances drainage (surgical or radiological). Most guidelines support drainage of pyogenic liver abscesses where possible. There are a number of options; radiologic guided needle aspiration or catheter drainage, or surgical drainage. Surgical drainage is usually reserved for those that are inaccessible by the percutaneous route. Both catheter drainage and intermittent needle aspiration appear to be effective for small abscesses in two randomized controlled trials [5,6]. One randomized trial found catheter drainage to be superior for abscesses over 10 cm [7], however two thirds of the patients in this study had amoebic abscesses. There are no randomized controlled trials comparing drainage with no drainage, but observational data has shown that patients with abscesses over 5 cm that are not drained have worse outcomes [8]. Abscesses under 5 cm that were not drained did not have worse outcomes.

The intravenous (IV) phase of antibiotic management usually consists of either IV ceftriaxone or cefazolin (with or without concomitant gentamicin). A retrospective observational study of 107 patients in Taiwan in 2003 found higher rates of complications (metastatic infection and death) in the 59 patients in the cefazolin group, compared to the 48 patients treated with an extended-spectrum cephalosporin (36.7% versus 6.3% respectively) [9]. Within the cefazolin arm there were higher rates of severe outcomes in the nine patients not receiving concomitant gentamicin, but this failed to reach significance. In 2008, another retrospective series of 110 patients was reported from Taiwan wherein 95% of the patients received cefazolin plus gentamicin [10]. The reported low rates of metastatic complications of 4.3% and mortality of 5.4% in those treated with cefazolin plus gentamicin appear at odds with the high rates seen in the cefazolin arm of previous study. A randomized controlled trial is due to be completed soon in Taiwan, comparing moxifloxacin 400 mg IV daily for 14 days, then 400 mg *per orale* (po) daily for 7 days, with ceftriaxone 2 g IV 12 hourly for 14 days, then oral cephalexin 1 g six hourly for 7 days. The study includes all pyogenic liver abscesses (although in Taiwan the majority will be *K. pneumoniae*) [11]. This is a pilot study, aiming to enroll 48 patients by April 2012. Whilst both arms complete the same duration of IV antibiotics, moxifloxacin has a bioavailability of 90%, so if both drugs are shown to be equivalent, this study may add support to earlier conversion to orals.

Currently, there is considerable variation in practice with regard to total antibiotic duration and more specifically the duration of IV antibiotics. There is a lack of evidence to guide practice in this area which our study aims to definitively address. A retrospective study by Ng in Hong Kong in 2002 identified 112 patients with pyogenic liver abscess (most commonly *K. pneumoniae*) and grouped them into two groups based upon their management [12]. Group 1 was those that received IV antibiotics for their whole treatment course, and group 2 were stepped down to oral antibiotics once clinically stable. Group 1 received on average 5.9 weeks of IV antibiotics. Group 2 received three point two weeks of IV and two point nine weeks of oral. There were three deaths in the group receiving continuous IV therapy, and none in the early step-down group. No statistically significant difference in outcomes was found, and no patients relapsed. The retrospective nature of this study limits the interpretation, because allocation to the two groups was not random so there is likely to be significant selection bias. While the study was not powered to show non-inferiority, there is no suggestion of a trend towards worse outcomes in those receiving shorter courses of IV antibiotics. This adds support to the need for prospective randomized studies looking at shorter durations of IV antibiotics for KLA.

There is one randomized controlled trial that has attempted to address the question of when to convert to oral antibiotics, conducted in Taiwan [13]. Any liver abscess was eligible, but the vast majority were *K. pneumoniae* monoinfection. The study was an open label randomized controlled trial, designed as a pilot study. The study arm was oral fleroxacin (a fluoroquinolone) for three weeks, starting from the day of enrollment (patients who had already been on antibiotics for 48 hours were excluded). The control arm was IV cefazolin 1 g eight hourly plus gentamicin 1 mg/kg eight hourly for two weeks, followed by oral cephalexin 1 g six hourly one to two weeks. Exclusion criteria were: pregnancy, breastfeeding, allergy to study drugs, glomerular filtration rate (GFR) < 30 ml/min, neutrophils < 0.5×10^3^/μl, prior treatment with effective antibiotic within 48 hours of enrollment, organisms resistant to study drugs. Sixty-one patients were enrolled. After exclusions 44 were followed-up. The endpoints were bacteriologic cure (70% in the oral arm versus 81.8% in the IV arm), clinical cure two weeks post completion (60% in the oral arm versus 81.8% in the IV arm). There was one death in each arm. This study has a number of limitations. Neither endpoint was adequately defined in the study. The study was not powered for non-inferiority. Patients in the control arm were reviewed on the ward every day, whilst patients in study arm were discharged home with less frequent medical input. The control arm received cefazolin, which is possibly not the most efficacious comparator [9]. Despite the limitations, this study highlights the need for a well designed randomized-controlled trial powered to show non-inferiority.

Currently, no local guidelines exist and there is wide variation in clinical practice. Based only upon the limited studies mentioned above, a number of organizations have written recommendations for the management of pyogenic liver abscess. These do not specifically address KLA and give variable advice to clinicians regarding the optimal time to step-down to oral therapy. The Australian Therapeutic Guidelines recommend 'After clinical improvement, consider a step-down to oral therapy. The total treatment duration should be four to six weeks’ [14]. The BMJ Best Practice Guidelines recommend that 'Parenteral therapy is given initially. If the patient is improving and fever and leukocytosis have resolved, the patient can be switched to an oral anti-infective regimen, typically for four to six weeks’ [15]. UpToDate recommends a total of four to six weeks, with IV antibiotics for the first two to three weeks if drained, or four to six weeks if not drained [16]. The results of our study will provide an evidence base for local and international guidelines.

### Preliminary studies

A prospective cohort study of all cases of KLA treated from 2005 to 2011 at two outpatient parenteral antibiotic therapy (OPAT) centers in Singapore (National University Hospital (NUH) and Tan Tock Seng Hospital (TTSH)) was completed to assess the safety and efficacy of OPAT for KLA [8]. One hundred and nine patients were enrolled. All patients had computed tomography (CT) or ultrasound (US) suggestive of a liver abscess, and either a positive blood culture or liver abscess fluid culture for *K. pneumoniae*. There were no deaths, or relapses post antibiotics. This study showed: mean duration of hospital stay of 15 days (range 2 to 84); mean duration of OPAT stay of 16 days (range 2 to 54); mean total time on IV antibiotics 31 days (range 11 to 98); and mean total oral antibiotics 6 weeks (range 0 to 21 weeks). Clinical response rate at four weeks was 73% and clinical cure one month after stopping antibiotics was 100% (using the same definitions as proposed in the section on endpoints below). Using discharge data from NUH over the same period, 196 patients met a diagnosis of KLA. This implies many patients did not reach OPAT, possibly due to logistical/financial reasons, but possibly also for medical reasons. Some would have remained inpatient at NUH, some would have been discharged to step-down care, and possibly some would have died. Clearly there is inherent selection bias in the above study because only patients well enough to be discharged to OPAT were included.

### Aims

Our study’s primary aim is to compare the efficacy of four weeks of IV antibiotics to early step-down to oral antibiotics in patients with KLA. Our results would help inform local and international practice guidelines regarding the optimal antibiotic management of KLA. A finding of non-inferiority in terms of clinical efficacy may translate to the wider adoption of a more cost-effective strategy that reduces hospital length of stay and improves patient-centered outcomes and satisfaction.

### Hypothesis

The hypothesis for our trial is that the degree of inferiority of our intervention (early step-down to oral antibiotics) to our active control (continued IV antibiotics) is no greater than 12%.

### Objectives

The primary objective is to compare the rate of clinical cure after four weeks of IV antibiotics to early step-down to oral antibiotics in patients with *Klebsiella pneumoniae* liver abscess, with clinical cure determined at Week 12 post randomization, and defined as CRP < 20 mg/l, plus absence of documented fever ≥ 38°C in the preceding seven days, plus most recent abdominal imaging showing that the maximal diameter of the abscess has reduced. The main secondary objective is to compare the rate of clinical response between the two study arms, with clinical response determined at Week 4 post-randomization, and defined as per clinical cure. Other secondary objectives are to compare the rate of the following during the study period, between the two study arms (assessed at 12 weeks): all cause mortality; unplanned readmission for any cause; unplanned need for drainage; metastatic complications; recrudescent or breakthrough *K. pneumoniae* bacteremia; need to escalate antibiotics due to worsening infection or secondary infection; vascular catheter complications. Additionally, to compare the length of hospital stay (from the date of randomization to the end of inpatient stay), length of time the subject requires medical leave following hospital discharge (censored at 12 weeks), total cost, subject quality of life (QoL), and adherence to study drug between the two study arms.

## Methods/design

### Study participants

The target number of subjects to be recruited is 152 over two years, across three academic teaching hospitals in Singapore (National University Hospital, Tan Tock Seng Hospital, and Singapore General Hospital). There is no restriction on subject recruitment by race or by gender. Pregnant and lactating women will be excluded from the study. Subjects will be identified by a research assistant (RA) who will be notified daily by radiology of all CT and US scans suggestive of liver abscess and by microbiology of all blood and fluid cultures positive for *K. pneumoniae*. The RA will screen eligible patients against study inclusion and exclusion criteria, and contact a study investigator to obtain informed consent. If the patient consents, the infectious diseases (ID) team will either take over management or co-manage the patient (with the primary physician’s agreement) to ensure that all elements of the protocol are adhered to. If the patient does not consent, the ID team will continue to provide consultation services.

### Inclusion criteria

1) Inpatient at time of enrollment.

2) Age ≥ 21 years.

3) CT or US scan within the preceding seven days suggestive of a liver abscess, as defined by presence of one or more focal areas of hypo- or hyper-attenuation within the liver.

4) *K. pneumoniae* isolated from abscess fluid or blood collected within the preceding seven days.

5) Able and willing to give informed consent.

### Exclusion criteria

Subjects meeting any of the following exclusion criteria at baseline will be excluded from participation:

1) Polymicrobial abscess - additional organisms isolated from blood or abscess fluid within the preceding seven days

2) 

a) *K. pneumoniae* resistant to ceftriaxone AND ertapenem

a) *K. pneumoniae* resistant to ciprofloxacin AND trimethoprim/sulfamethoxazole

3) On effective* IV antibiotics > seven days

4) 

a) Hypersensitivity to cephalosporins AND carbapenems; as defined by history of rash, urticaria, angiodema, bronchospasm or circulatory collapse following prior administration

a) Hypersensitivity to fluoroquinolones AND sulpha drugs; as defined by history of rash, urticaria, angiodema, bronchospasm or circulatory collapse following prior administration

a) History of penicillin anaphylaxis (angiodema, bronchospasm or circulatory collapse). Subjects with a history of only rash or urticaria or unknown reaction to penicillin can be included.

5) Inability to take oral medications for any reason

6) Severe sepsis or septic shock defined as unresolved hypotension (mean arterial pressure (MAP) < 70 mmHg) or tachycardia (heart rate (HR) > 110/min), or requirement of inotropic support or ventilation at time of eligibility. Should the subject’s hypotension or tachycardia subsequently resolve, and they cease to require inotropes and ventilation within seven days, they may be reconsidered for eligibility

7) Established endophthalmitis at time of screening (patients with visual symptoms should have ophthalmology review prior to enrollment)

8) Established central nervous system abscess at time of screening (patients with focal neurology should have cranial CT prior to enrollment)

9) Women who are pregnant or breastfeeding

10) Inability to obtain consent from subject

11) Patients on tizanidine or theophylline

12) Patients on concomitant drugs that can result in prolongation of the QT interval (for example, class IA or class III antiarrhythmics) or with risk factors for torsade de pointes (for example, known QT prolongation, uncorrected hypokalaemia)

13) Patients whose *K. pneumoniae* tests resistant to ciprofloxacin, and those with contraindications to ciprofloxacin will be tested for glucose-6-phosphate dehydrogenase (G6PD) deficiency, and excluded if deficient

14) Severely immunocompromized (for example, active leukemia or lymphoma, generalized malignancy, aplastic anemia, solid organ transplant, bone marrow transplant within two years of transplantation, or transplants of longer duration still on immunosuppressive drugs or with graft-versus-host disease, congenital immunodeficiency, current radiation therapy, HIV/AIDS with CD4 lymphocyte count < 200 and patients or on immunosuppressant medications)

15) Creatinine clearance < 15 ml/min

*defined as antibiotics to which the *K. pneumoniae* isolate in blood or abscess fluid is susceptible.

### Withdrawal criteria

A patient may be withdrawn from the study at any point if he or she withdraws consent, or if the investigator deems it is in the best interest of the patient or due to safety concerns (adverse reactions due to study drug, better treatment option available, and so on). Any patient withdrawn from the study intervention would remain in follow-up for observation of safety and efficacy endpoints. However, all patients are free to withdraw completely from the study at any time, without giving a reason. Should they elect to leave the study then they will default to standard of care, which is IV ceftriaxone 2 g daily for four weeks followed by oral ciprofloxacin for two weeks. Patients already randomized to the oral treatment arm may elect to stay on oral antibiotics for the remainder of their allocated treatment duration. Subjects who drop out will not be replaced. Any patient withdrawn from the study intervention would remain in follow-up for observation of safety and efficacy endpoints. They would be asked to return for the Week 12 assessment. If they decline, then they will be followed as per institutional routine practice - usually two weeks after completion of antibiotics.

### Study design

**Figure 1:**
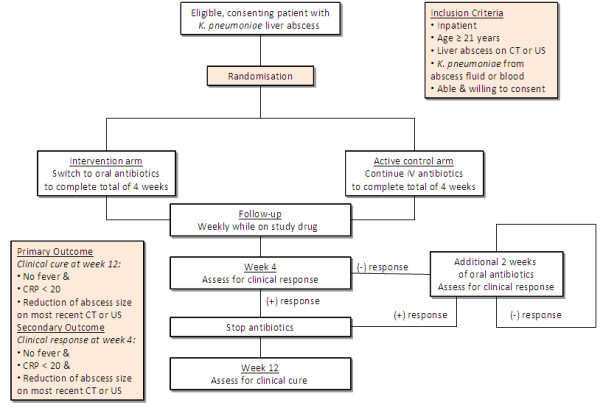
Trial entry, randomization and treatment flow diagram.

This study is a multi-center randomized open-label active comparator-controlled non-inferiority trial. Subjects fulfilling the inclusion/exclusion criteria are randomized to one of two arms: intervention arm; or active control arm, as shown in Figure 1. Participants randomized to the active control arm will receive IV ceftriaxone 2 grams daily (or ertapenem 1 gram daily if resistant) to complete a total of four weeks of IV antibiotics until the assessment at Week 4 post randomization. Patients can be discharged to OPAT at any point post randomization if clinically well, at the discretion of the treating physician/ID team. Patients discharged to step-down care will be followed up in OPAT clinic at the same weekly intervals as patients discharged to home. Patients will be assessed at Week 4 for clinical response. If they do not meet criteria for clinical response (as defined in the section on endpoints) then they will receive a further two weeks of oral ciprofloxacin 750 mg twice daily (or trimethoprim/sulfamethoxazole 5 mg/kg twice daily if resistant) and then re-reviewed (see below).

Participants randomized to the intervention arm will be immediately converted to oral ciprofloxacin 750 mg twice daily (or trimethoprim/sulfamethoxazole 5 mg/kg twice daily if resistant). Oral antibiotics will be continued until the Week 4 assessment. Patients can be discharged home at any point post randomization if clinically well, at the discretion of the treating physician/ID team. Patients will be followed-up weekly in OPAT to ensure the same follow-up as patients in the control arm. Patients will be assessed at Week 4 for clinical response. If they do not meet criteria for clinical response (as defined in the section on endpoints) then they will receive a further two weeks of oral ciprofloxacin 750 mg twice daily (or trimethoprim/sulfamethoxazole 5 mg/kg twice daily if resistant) and then re-reviewed (see below).

Baseline as well as serial clinical and laboratory data will be collected as per the trial schedule. Patients will be monitored daily by their clinical team in the hospital until discharge, with weekly assessments by the study team, and weekly study assessments as outpatients after discharge until the Week 4 assessment. If participants do not meet criteria for clinical response at Week 4 there will be a further Week 6 appointment at the end of the two-week extension of oral antibiotics. Patients not satisfying the criteria for clinical response after Week 6 will receive two-weekly extensions of oral antibiotics and two weekly visits, until they meet criteria for response. All participants will be asked to return to a follow-up assessment at Week 12 (all follow-up assessments will have a window of +/- three days).

### Randomization

After informed consent is signed, randomization into intervention or active control arms will be performed with a 1:1 allocation ratio. Balanced treatment assignments will be achieved using permuted block randomization, stratified by:

1) Abscess drainage (yes/no);

2) *K. pneumoniae* resistant to first-line agents (ceftriaxone or ciprofloxacin) (yes/no);

3) Study site.

The block length is determined by the study statistician and will not be made known to the clinical investigators or site personnel. All randomization will go through SCRI. Randomization will be done via direct web randomization. Authorized study center personnel will randomize the patient via a password protected internet website. The randomization system will then determine the treatment arm and provide the subject number to be used for the patient. The site monitor/clinical research associate (CRA) will be informed immediately in the event that the web randomization is not successful. Back-up randomization opaque envelopes (and related procedures) will also be prepared and provided to the site in case of internet failure.

Study arm allocation will not be blinded.

### Study visits and procedures

Please refer to trial schedule (Table 1) for overview of study procedures. Randomization will occur at the screening visit assuming the eligibility criteria have been met and informed consent has been obtained. If a patient has not had a blood culture drawn within the preceding seven days, this will be taken at the screening visit and the patient must wait for the result of the test prior to randomization (to ensure exclusion criterion 1 is met). Study drug will be commenced immediately post-randomization. The screening visit will include the following procedures: written informed consent; documentation of demographic data; full medical history including comorbidities, current medications, presenting and current symptoms; physical examination, including measurement of blood pressure, heart rate, temperature, and cardiovascular, respiratory and abdominal examination; baseline blood tests (full blood count (FBC), renal panel 1 (RP1), liver function tests (LFT), C-reactive protein (CRP), procalcitonin); urine pregnancy test; culture result and antibiotic sensitivities of the blood culture +/- abscess fluid culture; radiographic findings; antibiotic history since admission; whether abscess drainage was performed or is planned; World Health Organisation Quality of Life BREF questionnaire (WHOQOL-BREF). Patients whose *K. pneumoniae* tests resistant to ciprofloxacin and those with contraindications to ciprofloxacin will be tested for G6PD deficiency, and excluded if deficient. The number of days on effective IV antibiotics will be documented (effective defined as any IV antibiotic to which the isolate is susceptible). All patients will be referred to ophthalmology for dilated eye exam to screen for endophthalmitis as per current clinical practice at some institutions. Screening for other metastatic infectious complications will be undertaken only if symptoms or examination findings are suggestive. Study specific procedures will not be conducted until after informed consent has been obtained. Patients will also be asked during the informed consent process regarding storage of residual blood/abscess fluid specimens for future use in further elucidating host/pathogen factors in KLA syndrome.

**Table 1:** Trial schedule

**Assessments**	**Pre-enrolment (routine care)**	**Screening**	**Once between day 0 and day 7**	**Day 2–3, rpt every 2-3d until negative**	**Any point in study period if temp ≥38°C and any other secondary infection suspected**	**Daily**	**Every 7 days (+/- 3d)**	**Day 28 (+/- 3d)**	**Day 42 (+/- 3d) *(if oral continued)***	**Day 56 (+/- 3d) *(if oral continued)***	**Day 70 (+/- 3d) *(if oral continued)***	**Day 84 (+/- 7d)**
Informed consent		X										
Randomisation		X										
Demographics		X										
Past medical history^1^		X										
Antibiotic history		X^2^					X^3^	X^3^	X^3^	X^3^	X^3^	X^3^
Symptom assessment^4^		X					X	X	X	X	X	X
Temperature		X				X^5^	X^6^	X	X	X	X	X
Physical examination^7^		X					X	X	X	X	X	X
Screen metastatic complications^8^		X					X	X	X	X	X	X
Adverse event monitoring							X	X	X	X	X	X
Adherence check							X	X	X	X	X	X
Ophthalmology assessment			X									
FBC		X^9^					X	X	X	X	X	X
RP1		X^9^					X	X	X	X	X	X
LFT		X^9^					X	X	X	X	X	X
CRP		X^9^					X	X	X	X	X	X
Procalcitonin		X^9^					X	X	X	X	X	X
Urine pregnancy test		X^9^										
G6PD		X^10^										
Blood cultures	+/-	X^11^		X	X							
Chest X-ray		X^11^										
Abdominal CT or US	X^12^							X^13^		X^14^		X^15^
Abscess drainage^16^	+/-											
Abscess fluid culture^16^	+/-											
Study drug						X						
Subject diary of healthcare expenses						X						
Quality of Life Survey		X						X				X

^1^Full medical history including comorbidities, current medications, drug allergies, presenting and current symptoms. ^2^Document all antibiotics taken during this treatment episode up until enrollment. ^3^Include study drug and any additional antibiotics given. ^4^Standardized checklist. ^5^Self recorded daily temperature recording at home post discharge until Day 28 or until meeting criteria for clinical response. ^6^Self recorded weekly temperature recording at home after meeting criteria for clinical response, with weekly reminder call from the study team. ^7^Measurement of blood pressure, heart rate, and cardiovascular, respiratory and abdominal examination. ^8^Screen consists of standardized checklist of symptoms and signs, which if present trigger relevant investigations. ^9^If not already done in previous 48 hours. ^10^Only in patients whose *K. pneumoniae* tests resistant to ciprofloxacin, and those with contraindications to ciprofloxacin. ^11^If not already done in previous seven days. ^12^Scans must be within the preceding seven days. ^13^Day 28 scan to be performed within three days prior to the Day 28 visit. ^14^Day 56 scan to be performed within three days prior to the Day 56 visit, only in those patients failing to meet criteria for clinical response at day 42. ^15^Day 84 scan to be performed within three days prior to the Day 84 visit, only in those patients failing to meet criteria for clinical response at day 70. ^16^At any point during the current treatment episode.

Patients will be reviewed every seven days whilst inpatient. Assessment will consist of a standardized clinical assessment consisting of temperature, blood pressure, heart rate, symptom assessment and clinical examination. Blood tests (FBC, RP1, LFT, CRP, procalcitonin) will be performed a minimum of once every seven days while in hospital. In addition, temperature will be measured daily for all participants until Day 28 or meeting criteria for clinical response, whichever is longest. While inpatient or in OPAT this is part of routine clinical care; and for patients on the intervention arm (oral antibiotics), patients will be given thermometers to use daily after hospital discharge. Repeat blood cultures will be performed at day two to three post randomization on all patients with a positive blood culture at/prior to enrollment. If this remains positive it will be repeated every two to three days until negative. This is to ensure clearance of bacteremia.

Following discharge, participants in both arms of the study will be reviewed weekly in the OPAT clinic with a standardized clinical assessment consisting of temperature, blood pressure, heart rate, symptom assessment, clinical examination and blood tests (FBC, RP1, LFT, CRP, procalcitonin) as well as an adherence check for both study arms (pill count for subjects on oral; OPAT nurse documentation and infusion bottle count for subjects on IV). Within the three days preceding the Week 4 visit, all study participants will undergo follow-up abdominal imaging (CT or US) as per current standard of care in these cases. If participants do not meet criteria for clinical response at Week 4, there will be a further Week 6 appointment at the end of the two week extension of oral antibiotics. Patients not satisfying the criteria for clinical response after Week 6 will receive two weekly extensions of oral antibiotics and two weekly visits, until they meet criteria for response. Patients not meeting criteria for clinical response by the Week 6 visit will have a repeat abdominal scan (CT or US) arranged for within three days prior to the Week 8 visit. Patients not meeting criteria for clinical response by the Week 10 visit will have a repeat abdominal scan (CT or US) arranged for within three days prior to the Week 12 visit. There is no upper limit to the duration of antibiotics, but subjects will not be followed up beyond Week 12 within the study. Patients not meeting criteria for clinical response by the Week 12 visit will have failed to meet the primary endpoint of clinical cure, and will thereafter revert to routine clinical care.

All participants will return to a follow-up assessment at Week 12. This assessment will follow the same format as the preceding visits, with standardized clinical assessment consisting of temperature, blood pressure, heart rate, symptom assessment, clinical examination and blood tests (FBC, CRP, and procalcitonin).

The following direct and indirect costs over the whole study period will be collected from administrative sources where possible, and by self-report otherwise. Patients will be asked to maintain a diary of health care-related utilization and expenditures as well as to retain all related receipts and bills collected during the period of the study.

1) Inpatient treatment (hospital cost and billing systems for procedures, diagnostics and pharmaceuticals, verified against patient hospital bill): inpatient stay cost, diagnostics, supplies, antibiotics required for study treatment, other medications outside study treatment.

2) Outpatient care (patient diary, verified against copies of bills and receipts where possible): consultations at outpatient clinic, polyclinic, general practitioner, emergency department visit, ambulance service, care at community-based facilities.

3) Outpatient medication and supplies (patient diary, verified against copies of bills and receipts where possible): prescriptions; over the counter medication; other equipment and supplies.

4) Caregiver time (patient diary): hours spent by caregiver and identity of caregiver: whether professional or working/nonworking relative.

5) Patient time (patient diary): hours spent in treatment and additional days of work absenteeism.

6) Transport costs (patient diary, verified against copies of bills and receipts where possible): hours spent in transit to facilities for follow-up/other care, cost of transportation.

### Study endpoints

The primary endpoint is 'clinical cure’, determined at Week 12 post-randomization, and defined as CRP < 20 mg/l, plus absence of documented fever ≥ 38°C in the preceding week, plus most recent abdominal imaging showing that the maximal diameter of the abscess has reduced.

The main secondary endpoint is 'clinical response’, determined at Week 4 post-randomization, and defined as CRP < 20 mg/l, plus absence of documented fever ≥ 38°C in the preceding week, plus most recent abdominal imaging showing that the maximal diameter of the abscess has reduced. Other secondary endpoints (assessed at Week 12) are:

• all-cause mortality at any point between randomization and Week 12;

• unplanned readmission for any cause at any point between hospital discharge and Week 12;

• unplanned need for drainage after enrollment at any point between randomization and Week 12 (the screening visit will document any plans for elective drainage);

• metastatic complications occurring at any point between randomization and Week 12;

• new *K. pneumoniae* bacteremia occurring at any point between the first negative blood culture, and Week 12, with the same strain of *K. pneumoniae* as the original blood culture or abscess fluid culture determined by antibiotic susceptibility pattern);

• length of hospital stay (from the date of randomization to the end of inpatient stay, censored at Week 12);

• length of time the subject requires medical leave following hospital discharge (censored at Week 12);

• subject quality of life as defined by the WHOQOL-BREF assessed at Week 4 and Week 12 post-randomization;

• overall cost of each treatment strategy from the payer and total societal perspective for the course of the study until the final 12 Week follow-up;

• level of adherence during the entire study period, assessed at 12 weeks. Subject deemed to be compliant if 80% or more of prescribed antibiotics have been taken.

Safety endpoints include: need to stop study drug due to toxicity; toxicity as defined by Common Terminology Criteria for Adverse Events version 4.03 [17]; all adverse events (occurrence of adverse events up to 12 weeks post randomization or 30 days post last study dose if later than 12 week visit); serious adverse events (occurrence of serious adverse events at any time during the study period, whether or not they are thought to be related to the study drugs); need to escalate antibiotics due to worsening infection or secondary infection at any point between randomization and Week 12; vascular catheter complications at any point between randomization and Week 12.

### Study intervention

All study drugs and dosages are routinely used in clinical practice and will be ordered/dispensed as per each site’s institutional practice from the hospital pharmacy. The active control arm will receive IV ceftriaxone 2 g or ertapenem 1 g daily (adjusted for renal function) started immediately post randomization, and continued for four weeks from the date of randomization which is the standard dose used in clinical practice for complicated intra-abdominal infections including liver abscess. The intervention arm will receive oral ciprofloxacin 750 mg or trimethoprim/sulfamethoxazole 5 mg/kg twice daily (adjusted for renal function) started immediately post randomization, and continued for at least four weeks from the date of randomization. These antibiotics have been selected due to their prior extensive use in clinical practice with well-established safety and efficacy across a wide range of infections. They are also available as affordable generics. Ciprofloxacin is approximately 70% bioavailable. A 750 mg oral dose given every 12 hours has been shown to produce an area under concentration-time curve (AUC) at steady-state equivalent to that produced by an IV infusion of 400 mg given over 60 minutes every eight hours. A 750 mg oral dose results in a maximum measured plasma concentration (C_max_) similar to that observed with a 400 mg IV dose [18]. Trimethoprim/sulfamethoxazole is rapidly absorbed following oral administration. Peak blood levels for the individual components occur one to four hours after oral administration. During administration of 800 mg sulfamethoxazole and 160 mg trimethoprim twice a day, the mean steady-state plasma concentration of trimethoprim was 1.72 μg/mL which is well above the minimum inhibitory concentration (MIC) for susceptible Enterobacteriaceae including *K. pneumoniae*[19].

Ceftriaxone and ertapenem will be given IV using either peripheral cannula or a peripherally inserted central catheter (PICC) once daily. Administration may be done as inpatient, in OPAT or a step-down facility by healthcare staff, or at home by patients or their caregivers after training by OPAT staff (all these methods are currently in routine clinical use for patients on prolonged IV antibiotics). Ciprofloxacin and trimethoprim/sulfamethoxazole will be administered orally in tablets from each site’s pharmacy either as inpatient or at home. Patients on ciprofloxacin may not take tizanidine or theophylline or concomitant drugs that can result in prolongation of the QT interval (for example, class IA or class III antiarrhythmics).

### Safety considerations

'Unanticipated Problems Involving Risk to Subjects or Others’ (UPIRTSO) events and serious adverse events will be reviewed and classified by the site principal investigator (PI) or other investigator. Severity will be classified using a standard set of criteria for grading adverse events (Common Terminology Criteria for Adverse Events version 4.03). The relationship of the event to the study drug, and whether the event is an expected event or not, will be assessed using the listing of adverse effects contained in the summary of product characteristics for the antibiotics used. All SAEs that are unexpected and related to the study drug will be reported to the Health Science Authority (HSA).

A Data Safety Monitoring Board (DSMB) will be established, comprising two independent physicians and a statistician. An interim analysis - including both efficacy and safety endpoints will be performed after the first 50 subjects have completed 12 weeks of the study. The trial statistician will provide details of safety outcomes and any significant differences in efficacy according to treatment arm to the DSMB. These will be highlighted to the Trial Steering Committee along with the DSMB’s recommendations for action. If there is a significant safety concern raised by the, the DSMB may recommend to the PI that the trial should be stopped.

As stated in the Participant Information Sheet, complaints may be made to each site PI or the Domain Specific Review Board (DSRB). Complaints will be handled according to the normal procedures in operation in NUH, TTSH and SGH.

### Data collection and storage

The quality of all data collected will be regularly monitored by the site PI. Monitoring would be in accordance with Singapore Guidelines for Good Clinical Practice (SGGCP). The following elements will be monitored: recruitment proceeding as expected; cohort characteristics match the exclusion/inclusion criteria; deviations from protocol; timeliness, accuracy and confidentiality of all information in study documents and the central database. All study staff will be trained in the execution of study procedures including data collection and entry and training will be recorded in the study site file. For the purpose of this study, the electronic case report forms (eCRF) will serve as the source documentation. The data collected during the study will be entered into a central database by the study RA. The system will allow for audit tracking. Essential documents will be retained for a minimum of six years after the completion or discontinuation of the study.

### Determination of sample size

Whist a cohort OPAT study showed 100% cure, and another Singapore study failed to show any mortality from KLA [3,8], studies from Taiwan estimate a mortality of approximately 10% [4,10]. Given the inherent selection bias in the OPAT study, we estimate that overall 95% of patients will meet the primary endpoint of clinical cure in the control arm. The non-inferiority margin has been set at an absolute difference of 12% in the primary efficacy endpoint. This figure is justified because of the significant gains from use of oral antibiotics in terms of convenience and cost. The required sample size for a non-inferiority test for two binomial populations, with one-sided type 1 error 0.025, power 0.8, and non-inferiority margin 0.12, is 64 (in each arm) by use of the one-sided *z*-test with continuity correction. Assuming an attrition rate of 15%, a total of 152 patients would need to be recruited over two years.

### Statistical analysis

Data from all sites will be pooled for analysis. Descriptive statistics summarizing clinical observations data (for example, demographics) will be presented according to treatment arm. Continuous variables will be summarized using mean and standard deviation. Categorical variables will be summarized using the number of observations and percentages. All statistical analyses will be performed using SAS Version 9.3 or higher (SAS Institute Inc. 100 SAS Campus Drive, Cary, NC 27513–2414, USA; Phone:919-677-8000; Fax:919-677-4444). Statistical analysis and programming support will be provided by the Singapore Clinical Research Institute (SCRI).

An intention-to-treat (ITT) population set is defined as all randomized patients. The treatment group of patients in the ITT set is the planned treatment group, that is, according to the randomization list planned prior to the study commencement. A per protocol population is defined as all randomized patients who have taken at least one dose of study treatment and has the primary endpoint measured. The treatment group of patients in the per-protocol population is according to the treatment actually received. Patients will be assigned to the group as per the first dose received of the study treatment. If patients are issued the incorrect study treatment, they should continue on this treatment and not switch. For example if a patient was randomized to oral antibiotics but the first treatment they received after randomization was IV antibiotics they should continue on IV antibiotics. Statistical analysis for safety data and compliance will be carried out on an as-treated basis. This is the same as the per protocol population but without the requirement that the primary endpoint is measured. An as-treated set is defined as all randomized patients who have taken at least one dose of study treatment. The treatment group of patients in the as-treated set is according to the treatment actually received.

Demographic characteristics (age, gender and so on) and other baseline characteristics (for example, clinical measures taken at baseline) will be summarized by appropriated descriptive statistics according to randomization arm, that is, frequency and percent for categorical variables; mean, median, standard deviation and minimum/maximum for continuous variables.

The primary analysis will be conducted on both the per-protocol population and intention to treat population. The study will claim non-inferiority based on the results of the analysis on both the per protocol and ITT population. For the ITT, drop outs will be imputed as clinical cure if they meet all of the following criteria: last two CRPs show a downward trend or are < 20; last two recorded temperature measurements < 38°C; contact established via phone call at day 84 (+/- 7 days) and patient reports they are symptom free. If the criteria are not met then the case will be imputed as a clinical failure. The exact method will be used to test the hypothesis of non-inferiority for the pre-specified non-inferiority margin of 12%. A two-sided 95% confidence interval (CI) for the treatment difference will be calculated and compared with the equivalence margin (12%). If this 95% CI for the treatment difference excludes the non-inferiority margin (12%) then there is evidence of non-inferiority.

The Fisher’s exact test will be used to compare 12 week mortality between the two treatment arms and the associated exact 95% confidence interval will be calculated using the Clopper and Pearson method. Additionally we will calculate the time from baseline to death for each treatment group using the Kaplan-Meier product-limit method, censoring at the 12 week assessment, those that withdraw or are lost to follow-up during the study period will be censored at the point of last follow-up. Fisher’s exact test and the Clopper and Pearson CI will be calculated for all other binary endpoints between the two treatment arms. The Mann–Whitney *U-*test will be used to compare the duration of inpatient stay and the length of time the subject requires medical leave following discharge between the two treatment arms.

Quality of life data will be measured using the WHOQOL-BREF. The two overall items (overall perception of quality of life and overall perception of health) and the four domain scores (physical, psychological, social and environmental) will be examined. The Student’s *t*-test will be used to compare the change from baseline at both 4 weeks and 12 weeks for each of these scores between treatment arms, as well as the change between 4 weeks and 12 weeks. In further exploratory analysis we will fit a mixed model to examine the effect of time and treatment on each of the four domain scores.

Duration of treatment and treatment compliance will be summarized by arm. For each subject in the treated population, an assessment of overall percentage compliance will be calculated as the percentage of patients who received at least 80% of prescribed antibiotics, as per protocol dosage. Compliance will be considered unknown if it cannot be calculated because of missing data. The number of patients discontinuing randomized investigational product, as well as the reason for discontinuation, will be summarized and listed.

An economic analysis will be performed. Admission costs will be obtained by searching the hospital’s data systems. Information retrieved will include diagnoses, tests, procedures and so on, for the period that participants are inpatients. The price of the specific antibiotics dispensed to each participant will be obtained from the hospital pharmacy database system. Following discharge, direct and indirect costs of all study related medical or non-medical healthcare utilization will be tracked via patient diaries and recordkeeping, inclusive of travel costs, days of work lost and the value of caregiver time. No discounting of costs will be necessary given the time span. The direct and indirect costs associated with the alternative treatment arms used will be calculated per patient at 12 weeks. The costs will be summarized using mean and median values and presented according to treatment arm. The primary cost-effectiveness ratio to be calculated will be the total incremental cost per clinical cure at 12 weeks between the two groups. The Student’s *t*-test will be used to compare the incremental cost effectiveness ratio (ICER) as well as mean costs between the two treatment groups. The bootstrap technique will be used to obtain confidence intervals around the estimated ICER. We will generate an empirical sampling distribution for the ICER using trial data by generating 1,000 independent samples of 100 cost and effect pairs from both the groups’ data drawn with replacement and calculating the ICER each time. We will then report 95% bias-adjusted confidence intervals for the ICER. Anticipating that actual cost and resource-use data is likely to be missing, we will impute values for the missing cases if necessary. The analysis will be performed using costs computed from both analytical perspectives. We also anticipate potential uncertainty around some values and assumptions made for unit costs. In such instances, sensitivity analysis will be performed to further explore the robustness of our results to the baseline assumptions that are made.

The analysis of safety endpoints will be on the as treated population. Numbers and percentages needing to stop study drug due to toxicity will be summarized between the two treatment arms; as will the numbers and percentages of reported clinical symptoms of moderate/severe grade (by body systems). Numbers and percentages of patients with at least one adverse event up to 12 weeks post randomization or 30 days post last study treatment (whichever is last) will be tabulated by treatment group and details of the event will be summarized. The numbers and percentages of serious adverse events will be tabulated by treatment group and the details will be summarized. Numbers needing to escalate antibiotics due to worsening infection or secondary infection at any point between randomization and Week 12 will be compared between arms using the Fisher’s exact test and the Clopper and Pearson CI will be calculated.

## Discussion

### Ethical considerations

This protocol and the associated informed consent documents has be reviewed and approved by the NHG DSRB and Health Science Authority prior to initiation of study procedures.

Patients fulfilling the study inclusion criteria will be approached and briefed by a delegated study team member about all the pertinent aspects of the study. Consent will be obtained in a private, quiet room (such as the ward family conference room) where the subject will not be disturbed by other hospital staff/inpatients. The patient will be given sufficient time to ask any questions related to the study. Only after the patient has voluntarily agreed to participate in the study, will he/she be asked to document his/her approval by signing on the consent form. The consent will not be taken in front of the doctor managing the patient so that the patient does not feel coerced to give consent. Consent will be taken only by study team members (PI or co-investigators) who are authorized to do so by the PI in the study delegation log. Patients will be asked during the informed consent process regarding storage of residual blood/abscess fluid specimens for future use in further elucidating host/pathogen factors in KLA syndrome.

All study findings and documents will be regarded as confidential. The investigators and other study personnel must not disclose such information without prior written approval from the PI. Subject confidentiality will be strictly maintained to the extent possible under the law and as required by SGGCP. Subject names must not be disclosed. They will be identified on the CRFs and other study documents by their initials and assigned subject number. A record of the contact details will be kept separately by study personnel, as this is necessary for contacting participants to ensure adequate follow-up, which is an essential part of the study design. However, this information will be stored securely, and will be deleted six months after the completion of the trial.

## Trial status

• The trial has been granted ethics approval by National Healthcare Group (NHG) Domain Specific Review Boards (DSRB) (Approval ID: 2012/01035-AMD0001).

• The trial has been granted a Clinical Trials Certificate (CTC) by Health Science Authority (HSA) (Certificate number: CTC1300292).

• Funding has been approved by the National Medical Research Council (NMRC) (Application number: CNIG12nov013).

• The trial is listed on clinicaltrials.gov (Identifier: NCT01723150).

• Recruitment will start in October 2013.

## Abbreviations

A-KLASS: Antibiotics for Klebsiella liver abscess study; ALP: Alkaline phosphatase; ALT: Alanine aminotransferase; AST: Aspartate aminotransferase; AUC: Area under concentration-time curve; BMJ: British Medical Journal; CD4: Cluster of differentiation 4; CI: Confidence interval; CrCl: Creatinine clearance; Cmax: Maximum measured plasma concentration following each treatment; CRA: Clinical research associate; CRF: Case report form; CRP: C-reactive protein; CT: Computed tomography; DSMB: Data Safety Monitoring Board; DSRB: Domain Specific Review Board; eCRF: Electronic case report form; FBC: Full blood count; G6PD: Glucose-6-phosphate dehydrogenase; GFR: Glomerular filtration rate; HIV/AIDS: Human immunodeficiency virus infection/acquired immunodeficiency syndrome; HR: Heart rate; HSA: Health Science Authority; ICER: Incremental cost effectiveness ratio; ID: Infectious diseases; IRB: Institutional Review Board; ITT: Intention-to-treat; IV: Intravenous; K. pneumoniae: *Klebsiella pneumoniae*; KLA: Klebsiella liver abscess; LDH: Lactate dehydrogenase; LFT: Liver function test; MAP: Mean arterial pressure; MIC: Minimum inhibitory concentration; NA: Not applicable; NHG: National Healthcare Group; NUH: National University Hospital; OPAT: Outpatient parenteral antibiotic therapy; PI: Principal investigator; PICC: Peripherally inserted central catheter; po: *per orale*; QALY: Quality-adjusted life year; QT: Measure between Q wave and T wave in the heart’s electrical cycle; QOL: Quality of life; RA: Research assistant; RP1: Renal panel 1 (sodium, potassium, urea, creatinine); SAEs: Serious adverse events; SAS: Statistical Analysis Software; SCRI: Singapore Clinical Research Institute; SGGCP: Singapore Guidelines for Good Clinical Practice; TTSH: Tan Tock Seng hospital; UPIRTSO: Unanticipated Problems Involving Risk to Subjects or Others; US: Ultrasound; WHOQOL-BREF: World Health Organization Quality of Life BREF questionnaire.

## Competing interests

All authors report no competing interests.

## Authors’ contributions

JM was responsible for overall study design and drafted the manuscript. RP and MG participated in study design and developed the statistical analysis plan. JY developed the economic analysis plan. TT, DL and DF participated in study design and will participate in acquisition of data. SA conceived of the study, participated in its design and supervised the project. All authors read and approved the final manuscript.
